# Supplementary material for: Mouse Models of Achromatopsia in Addressing Temporal “Point of No Return” in Gene-Therapy
Source: Int J Mol Sci. 2021 Jul 28;22(15):8069. doi: 10.3390/ijms22158069 (PMC8347118; doi:10.3390/ijms22158069)

Supplementary Materials

Materials and Methods

Sequence "*floxed-miniSTOP*" cassette - 393 bps

Sequence of *floxed-miniSTOP* (393 bps). For clarity corresponding regions of each sequence are highlighted in different colors: loxP, Adenovirus splice acceptor segment 1 and 2, SV40 poly(A).

AACTAGTGGATCCGGAACCCTTAATATAACTTCGTATAGCATACATTATACGAAGTTATAAA
CCATGGTAGGGCGCAGTAGTCCAGGGTTTCCTTGATGATGTCATACTTATCCTGTCCCTTTTTT
TTCCACAGCTCGCGGTTGAGGACAAACTCTTCGCGGTCTTTCCAGTTAACTGATTAGGGCGCG
CCTTAATTAAAACTTGTTTATTGCAGCTTATAATGGTTACAAATAAAGCAATAGCATCACAAA
TTTCACAAATAAAGCATTTTTTTCACTGCATTCTAGTTGTGGTTTGTCCAAACTCATCAATGTA
TCTTATCATGTCTGGATCATAACTTCGTATAGCATACATTATACGAAGTTATTAGGTCCCTCGA
CCTGCAGCCCAAG

Sequence "*ssODN*" - 653 bases

Sequence of Single-stranded oligo donor (653 bases). For clarity corresponding regions of each sequence are highlighted in different colors: 5′ homologous (115 bases), floxed-miniSTOP (393 bases), 3′ homologous (145 bases).

AACACCTCGCCCTCACTCAGACATCAGCTCCATCCCTTCCTGTACTTGGATACTGTCCTTGGC
AATGCCAGCTTCAGCAAGAAGCTGAGGGAGATAGAGGCCATGATCGCAGTCCAACTAGTGG
ATCCGGAACCCTTAATATAACTTCGTATAGCATACATTATACGAAGTTATAAACCATGGTAG
GGCGCAGTAGTCCAGGGTTTCCTTGATGATGTCATACTTATCCTGTCCCTTTTTTTCCACAGC
TCGCGGTTGAGGACAAACTCTTCGCGGTCTTTCCAGTTAACTGATTAGGGCGCGCCTTAATTA
AAACTTGTTTATTGCAGCTTATAATGGTTACAAATAAAGCAATAGCATCACAAATTTCACAA
ATAAAGCATTTTTTTCACTGCATTCTAGTTGTGGTTTGTCCAAACTCATCAATGTATCTTATCAT
GTCTGGATCATAACTTCGTATAGCATACATTATACGAAGTTATTAGGTCCCTCGACCTGCAGC
CCAAGAAGAGGAAAAGATCTTCAGACTCAGAAGAATCATAAAATGATGAGGAGTCTCTAAG
CCAAGATGCTACACAAGGCACTCCATAGACACACGCACAAGCACTATAGCATCCATAGATC
TTGCACCTGGAGAGCCAATCAACCAGAT

Sequence "*floxed-STOP*" cassette - 2886 bps

Sequence of *floxed-STOP* cassette (2886 bps). For clarity corresponding regions of each sequence are highlighted in different colors: loxP, Splicing acceptors, SV40 poly(A).

CGCGGTCTTTCCAGTGGTTAATTAAATAACTTCGTATAGCATACATTATACGAAGTTATGGCC
AGCCTAAGCTAGAGTTTGAATGTAGCCTATACAAGACCCTGTCTCAAAAACCAAGCAAAAGT
AAAACCCCAGGAAACTGGGGGTTTGTATGCCTCTCCTGAACTAATTAATATCTATCTCCCCTT
CTTCATTTCCTTAAAGGAAGAATTCCTGTCCCTTTTTTTTCCACAGAATTCACCGACTGTGGTG
AATGGAATGTATGTCCTTTCCAGAACCTGGTCCACTAAGATACATTGATGAGTTTGGACAAA
CCACAACTAGAATGCAGTGAAAAAAATGCTTTATTTGTGAAATTTGTGATGCTATTGCTTTAT

TTGTAACCATTATAAGCTGCAATAAACAAGTTTACCACATTTGTAGAGGTTTTACTTGCTTTAA
AAAACCTCCCACACCTCCCCCTGAACCTGAAACATAAAATGAATGCAATTGTTGTTGTTGTA
AATATAAAATTTTTAAGTGTATAATGTGTTAAACTACTGATTCTAATTGTTTGTGTATTTTAGT
AAGATACATTGATGAGTTTGGACAAACCACAACTAGAATGCAGTGAAAAAAATGCTTTATTT
GTGAAATTTGTGATGCTATTGCTTTATTTGTAACCATTATAAGCTGCAATAAACAAGTTCTTAG
AGCTTTAAATCTCTGTAGGTAGTTTGTCCAATTATGTCACACCACAGAAGTAAGGTTCCTTCA
CAAAGATCCCTCGACCAGACATGAGTAAATATAAAATTTTTAAGTGTATAATGTGTTAAACT
ACTGATTCTAATTGTTTGTGTATTTTAGTAAGATACATTGATGAGTTTGGACAAACCACAACT
AGAATGCAGTGAAAAAAATGCTTTATTTGTGAAATTTGTGATGCTATTGCTTTATTTGTAACC
ATTATAAGCTGCAATAAACAAGTTCTTAGAGCTTTAAATCTCTGTAGGTAGTTTGTCCAATTA
TGTCACACCACAGAAGTAAGGTTCCTTCACAAAGATCCCTCGACCAGACATGAGAAGTTCCT
ATTCTCTAGAAAGTATAGGAACTTCGGGTAGGGGAGGCGCTTTTCCCAAGGCAGTCTGGAGC
ATGCGCTTTAGCAGCCCCGCTGGGCACTTGGCGCTACACAAGTGGCCTCTGGCCTCGCACAC
ATTCCACATCCACCGGTAGGCGCCAACCGGCTCCGTTCTTTGGTGGCCCCTTCGCGCCACCTT
CTACTCCTCCCCTAGTCAGGAAGTTCCCCCCCGCCCCGCAGCTCGCGTCGTGCAGGACGTGA
CAAATGGAAGTAGCACGTCTCACTAGTCTCGTGCAGATGGACAGCACCGCTGAGCAATGGA
AGCGGGTAGGCCTTTGGGGCAGCGGCCAATAGCAGCTTTGCTCCTTCGCTTTCTGGGCTCAGA
GGCTGGGAAGGGGTGGGTCCGGGGGCGGGCTCAGGGGCGGGCTCAGGGGCGGGGCGGGCG
CCCGAAGGTCCTCCGGAGGCCCGGCATTCTGCACGCTTCAAAAGCGCACGTCTGCCGCGCTG
TTCTCCTCTTCCTCATCTCCGGGCCTTTCGACCTGCAGCCTGTTGACAATTAATCATCGGCATA
GTATATCGGCATAGTATAATACGACAAGGTGAGGAACTAAACCATGGGATCGGCCATTGAA
CAAGATGGATTGCACGCAGGTTCTCCGGCCGCTTGGGTGGAGAGGCTATTCGGCTATGACTG
GGCACAACAGACAATCGGCTGCTCTGATGCCGCCGTGTTCCGGCTGTCAGCGCAGGGGCGCC
CGGTTCTTTTTGTCAAGACCGACCTGTCCGGTGCCCTGAATGAACTGCAGGACGAGGCAGCG
CGGCTATCGTGGCTGGCCACGACGGGCGTTCCTTGCGCAGCTGTGCTCGACGTTGTCACTGAA
GCGGGAAGGGACTGGCTGCTATTGGGCGAAGTGCCGGGGCAGGATCTCCTGTCATCTCACCT
TGCTCCTGCCGAGAAAGTATCCATCATGGCTGATGCAATGCGGCGGCTGCATACGCTTGATC
CGGCTACCTGCCCATTCGACCACCAAGCGAAACATCGCATCGAGCGAGCACGTACTCGGAT
GGAAGCCGGTCTTGTCGATCAGGATGATCTGGACGAAGAGCATCAGGGGCTCGCGCCAGCC
GAACTGTTCGCCAGGCTCAAGGCGCGCATGCCCGACGGCGATGATCTCGTCGTGACCCATGG
CGATGCCTGCTTGCCGAATATCATGGTGGAAAATGGCCGCTTTTCTGGATTCATCGACTGTGG
CCGGCTGGGTGTGGCGGACCGCTATCAGGACATAGCGTTGGCTACCCGTGATATTGCTGAAG
AGCTTGGCGGCGAATGGGCTGACCGCTTCCTCGTGCTTTACGGTATCGCCGCTCCCGATTCGC
AGCGCATCGCCTTCTATCGCCTTCTTGACGAGTTCTTCTGAGGGGATCAATTCTCTAGAGCTC
GCTGATCAGCCTCGACTGTGCCTTCTAGTTGCCAGCCATCTGTTGTTTGCCCCTCCCCCGTGCC
TTCCTTGACCCTGGAAGGTGCCACTCCCACTGTCCTTTCCTAATAAAATGAGGAAATTGCATC
GCATTGTCTGAGTAGGTGTCATTCTATTCTGGGGGGTGGGGTGGGGCAGGACAGCAAGGGGG
AGGATTGGGAAGACAATAGCAGGCATGCTGGGGATGCGGTGGGCTCTATGGCTTCTGAGGC
GGAAAGAACCAGCTGGGGCTCGACTAGAGCTTGCGGAACCCTTCGAAGTTCCTATTCTCTAG
AAAGTATAGGAACTTCATAACTTCGTATAGCATACATTATACGAAGTTATGGTACCGCGAGA
CCCACGCTCACCGGC

Figure S1. Electroretinography of three founder mice from *Easi*-CRISPR.
Photopic serial intensity ERG showed decreased *b*-wave amplitudes in #6 founder mouse (homozygous-like knock-in) compared to #2 (heterozygous-like knock-in) and #3 (no knock-in) founder mice. PCR results of these founder mice were showed in Figure 2C.

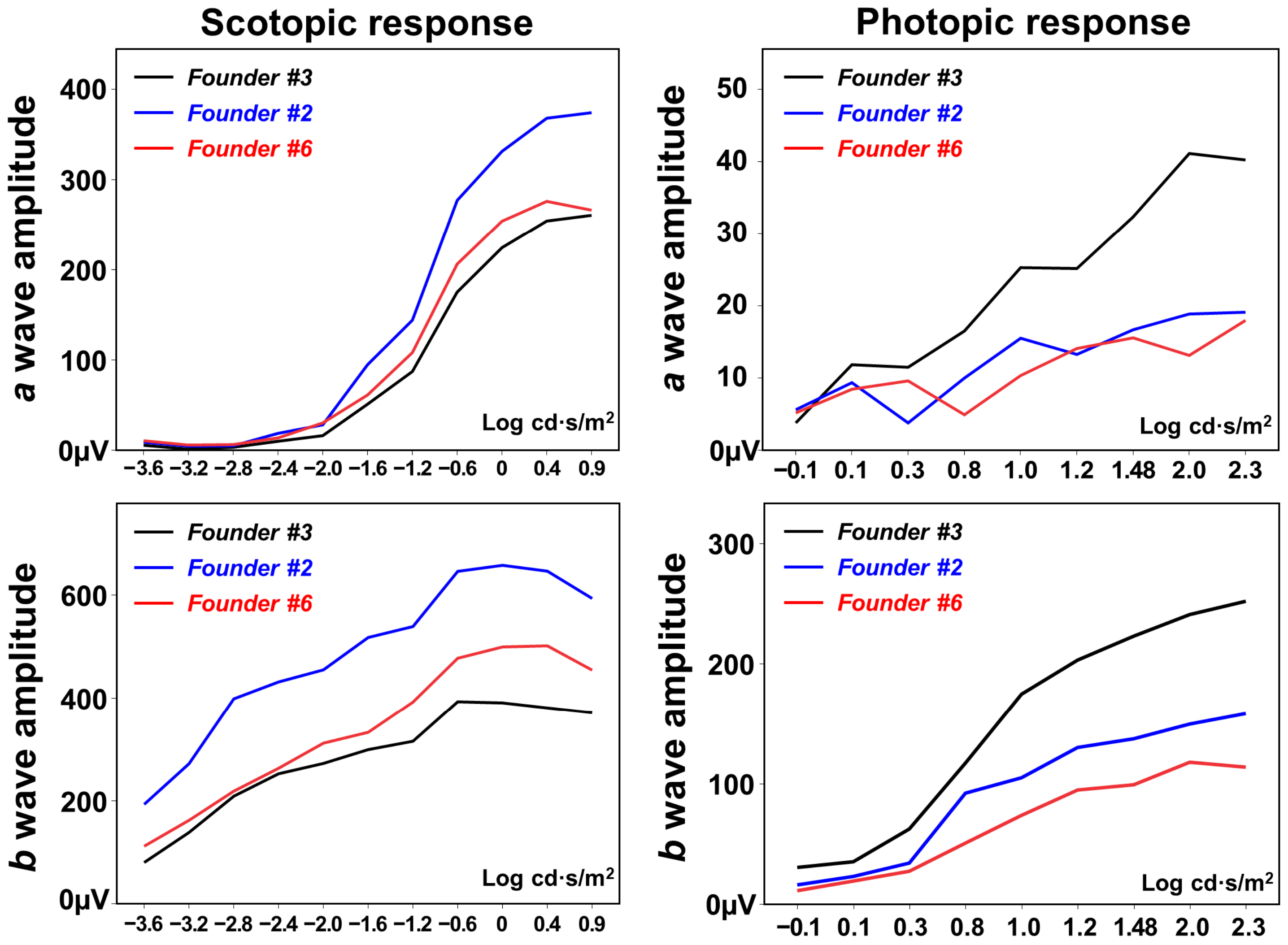

Figure S2. Histology of *Cnga3* $^{floxed\text{-}miniSTOP}$ mice at 6 months old

There was no difference in terms of outer nuclear layer between *Cnga3* $^{+/+}$, *Cnga3* $^{floxed\text{-}miniSTOP/+}$ and *Cnga3* $^{floxed\text{-}miniSTOP}$ mice from the same litter at 6 months old.

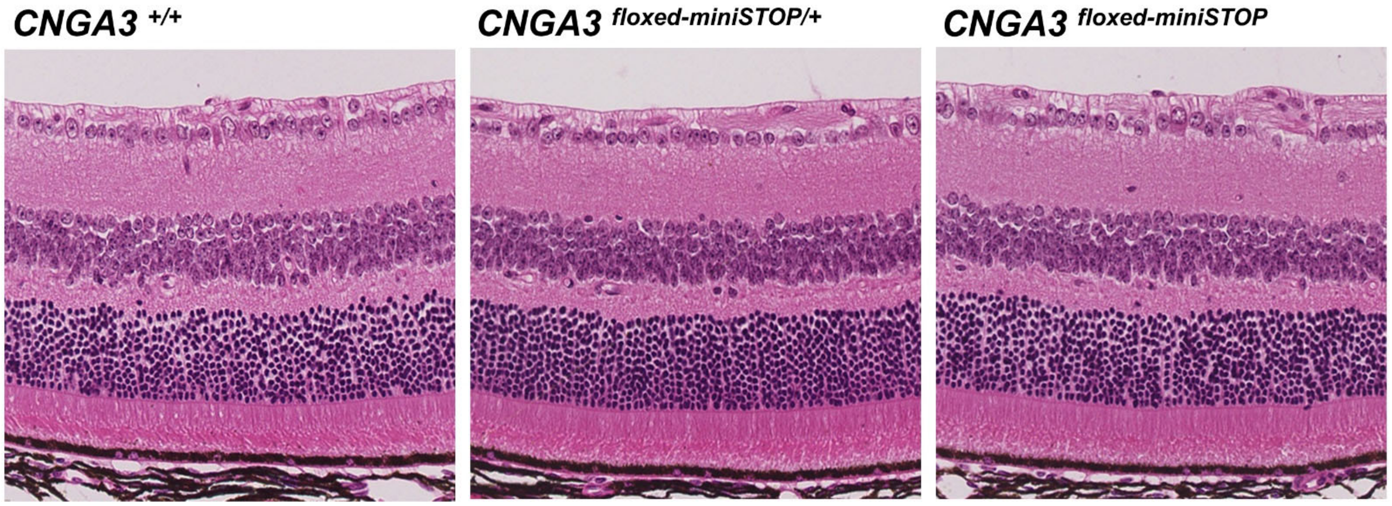

Supplement: Supplementary file 1 [file ijms-22-08069-s001.zip › ijms-1297742-supplementary.pdf]
